# Cost-effectiveness of edoxaban compared to warfarin for the treatment and secondary prevention of venous thromboembolism in the UK

**DOI:** 10.1080/20016689.2018.1495974

**Published:** 2018-07-17

**Authors:** Emilie Clay, Aurélien Jamotte, Peter Verhamme, Alexander T. Cohen, Ben. A. Van Hout, Pearl. Gumbs

**Affiliations:** a Health Economic Outcome Research​, Creativ-Ceutical, Paris, France; b Department of Cardiovascular Sciences, University of Leuven, Leuven, Belgium; c Department of Thrombosis and Haemostasis, Guy’s & St. Thomas’ Hospital, London, UK; d School for Health and Related Research, University of Sheffield, Sheffield, UK; e Market Access, Daiichi Sankyo Europe, Munich, Germany

**Keywords:** Edoxaban, warfarin, anticoagulant, venous thromboembolism, deep vein thrombosis, pulmonary embolism, economic evaluation, Markov model, cost-effectiveness

## Abstract

**Background**: Venous thromboembolism (VTE), which includes pulmonary embolism (PE) and deep vein thrombosis (DVT), is the third most common acute cardiovascular disease and represents an important burden for patients and payers.

**Objective**: The aim was to estimate the cost-effectiveness of edoxaban, a non-VKA oral anticoagulant vs. warfarin, the currently most prescribed treatment for VTE in the UK.

**Study design**: A Markov model was built using data from the Hokusai-VTE randomised controlled trial to estimate the lifetime costs and quality-adjusted life years (QALYs) in patients with VTE treated with edoxaban or warfarin over a lifetime horizon, from the UK National Health Services perspective. The model included VTE recurrences, VTE-related complications (post-thrombotic syndrome and chronic thromboembolic pulmonary hypertension), and several types of bleeds associated with anticoagulation treatment. Patients were treated during a period of 6 months after the first VTE event, followed by flexible treatment duration (from 6 months to lifetime) after recurrence, i.e., tertiary prevention.

**Results**: Edoxaban was found dominant vs. warfarin with 0.033 additional QALY and £55 less costs. The reduction of patient management costs, specifically monitoring costs, outweighed the higher drug costs. Edoxaban was dominant in all subgroups (index DVT only, all PE cases (PE with or without DVT), PE without DVT and PE with DVT). Cost-savings ranged from £54 to £81 while additional QALYs ranged from 0.031 to 0.046. Edoxaban was found dominant in 88.6% of cases and cost-effective in additional 10.9% of cases considering a £20,000 threshold in the probabilistic sensitivity analysis.

**Conclusion**: Edoxaban may improve patients’ quality of life in a lifetime horizon without additional costs for the healthcare system due to lower bleeding risk and no monitoring cost compared to warfarin.

## Introduction

Venous thromboembolism (VTE) is a potentially life-threatening disease that includes deep vein thrombosis (DVT) and pulmonary embolism (PE). VTE is characterised by the presence of a thrombus in the deep veins of the leg in cases of DVT, or in a pulmonary vessel in cases of PE. The predominant symptoms of DVT include pain, tenderness, and swelling of the involved limb, while those of PE include dyspnoea, tachypnoea, and pleuritic chest pain. Though DVT and PE have different manifestations, they are considered to be complementary manifestations of the same pathophysiological process. It has been observed that about 66% of patients present with DVT only, whereas the remaining present with PE [1]. VTE is a source of significant economic burden in Europe. The total number of symptomatic VTE events per year in Europe was estimated to be over 684,000 cases of DVT, over 434,000 cases of PE, and over 543,000 VTE-related deaths [2].

In the UK, pharmacotherapeutic treatment and prevention of acute DVT and PE consists of the use of different classes of anticoagulant medication. Current standard of care involves initial treatment with a parenteral anticoagulant (such as subcutaneous low molecular weight heparin (LMWH), unfractioned heparin (UFH), or fondaparinux), followed by a vitamin K antagonist (VKA), typically warfarin [3]. However, warfarin treatment has many disadvantages, such as a narrow therapeutic range, a need for careful dose adjustment, a need to carefully monitor diet and concomitant medication, and frequent monitoring of the international normalised ratio (INR) [4]. Non-VKA oral anticoagulants (NOACs) such as dabigatran, rivaroxaban, apixaban, and edoxaban have been recently recommended by the National Institute for Health and Care Excellence (NICE) for VTE treatment[5]. NOACs are clinically non-inferior to VKAs, with fewer and less serious side effects [6].

Edoxaban tosylate (Lixiana®) is a NOAC, which selectively and reversibly inhibits coagulation factor Xa directly, thus preventing the activation of prothrombin to thrombin. After initial treatment with heparin, the recommended dose for edoxaban is 60 mg once daily, with a dose reduction to 30 mg once daily for specific patient meeting predefined characteristics depending on: renal function, low body weight, and the use of P-gp inhibitors [7]. The Hokusai-VTE trial was a phase III, event-driven, randomised, double-blind, parallel-group, multi-centre, multi-national non-inferiority study designed to evaluate the benefits and risks of edoxaban compared to warfarin in reducing the risk of symptomatic recurrent VTE in patients with documented acute symptomatic DVT and/or PE. All patients received an initial therapy with LMWH (enoxaparin) or UFH, followed by either edoxaban (60mg/30mg dose reduced per day) or warfarin (dose adjusted to maintain INR between 2.0 to 3.0) in a double-dummy fashion. The primary efficacy outcome was symptomatic recurrent VTE, and the safety outcome was first major or clinically relevant non major bleeds. The Hokusai-VTE trial included 8292 adults with VTE, edoxaban was reported to be non-inferior for preventing recurrent VTE when compared to warfarin, and treatment with edoxaban vs. warfarin resulted in fewer bleeds [8].

The objective of this study was to develop a model to estimate the cost-effectiveness of edoxaban vs. warfarin in the treatment of and/or prevention of VTE in a UK setting from an NHS perspective.

## Materials and methods

### Model description

A Markov model was developed to simulate the therapeutic management of VTE patients, including the potential adverse events of treatment and disease-related complications. The model captured the impact of VTE on quality of life, resource utilisation, and associated costs over a lifetime horizon (50 years).

**Figure 1.**
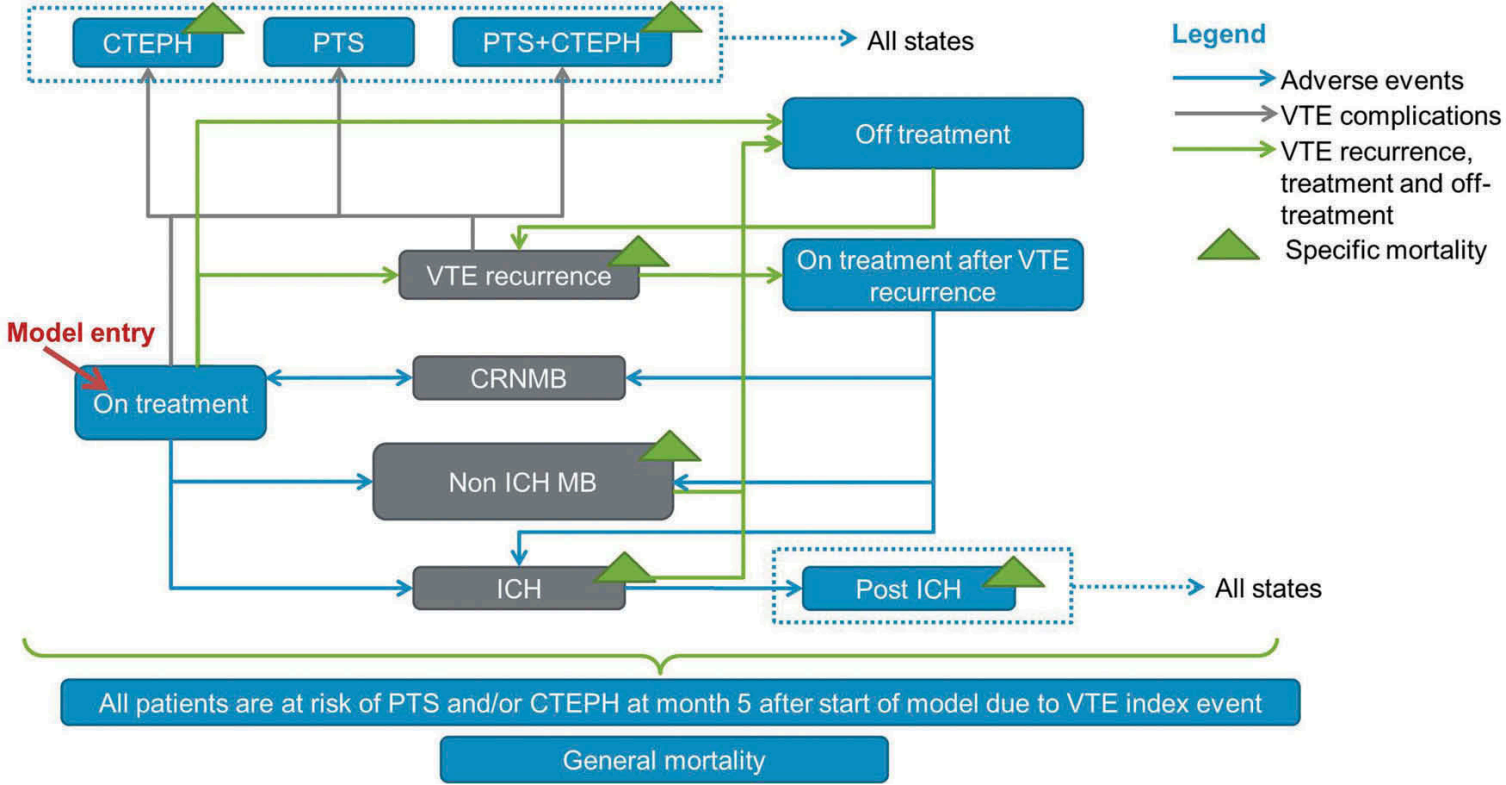
Structure of the Markov model.

A total of eleven health states were defined to describe the health outcomes and resource implications of VTE management (Figure 1). Eight health states were mutually exclusive: on treatment after an index VTE (iVTE), off treatment, recurrent VTE (rVTE), on treatment after an episode of rVTE, clinically relevant non-major bleed (CRNMB), intracranial haemorrhage (ICH), non-ICH major bleed (non-ICH MB), and death. Three health states, describing the long-term consequences of VTE, were defined as concomitant health states, i.e., a patient could experience one or more of the three following health states simultaneously with one of the eight health states described previously: severe post-thrombotic syndrome (PTS), chronic thromboembolic pulmonary hypertension (CTEPH), and disability following an ICH. The chosen cycle length was one month, enabling patients to transition between health states on a monthly basis.

The population included upon model entry was a cohort of adult (≥ 18 years) patients that had initiated treatment after an index VTE event (DVT and/or PE). Upon model entry, each patient received either 6.5 days of initial LMWH treatment followed by warfarin or 5 days of initial LMWH treatment followed by edoxaban for a duration of 6 months. There was a possibility for patients to experience an rVTE at the end of each cycle. Patients could only experience adverse events (CRNMB, non-ICH MB, and ICH) while on anticoagulant treatment. Patients were assumed to discontinue treatment following a major bleed, ICH, or non-ICH major bleeds. After an rVTE, i.e., tertiary prevention of VTE, patients who had both an index and recurrent PE (28% according to the Hokusai-VTE trial) were assumed to initiate a new anticoagulant strategy for a lifelong period, unless the patient would experience an MB. The other patients experiencing an rVTE were treated for 6 months. Death rates were based on a combination of age-specific general mortality rates and disease-specific mortality rates for patients suffering from an rVTE, non-ICH MB, ICH, disability following ICH or CTEPH.

The health outcomes included cumulative incidences for rVTE, adverse events and complications, life years and quality-adjusted life years (QALYs), average cumulative costs per treatment (on treatment costs, adverse events costs, total costs), the incremental cost-effectiveness ratio (ICER), and the net monetary benefit (NMB). The net monetary benefit was calculated according to the following equation: NMB = ΔE.λ-ΔC, where λ denotes the willingness-to-pay for a QALY gained, ΔE the incremental QALYs, and ΔC the incremental costs. The net monetary benefit represents the difference between how much one would be willing to pay for the additional QALYs gained by adopting the intervention compared to the alternative intervention costs. Finally, in accordance with NICE guidelines, an annual discount rate of 3.5% was adopted for costs and outcomes [9].

### Transition probabilities

The baseline probabilities of VTE recurrence and bleeds were derived from a post-hoc analysis of the Hokusai-VTE clinical trial [8] (Table 1). Similarly, the odds ratios of developing VTE recurrence and bleeds at baseline were based on the same analyses of the Hokusai-VTE trial data to estimate the time dependent probabilities. The baseline probabilities and odds ratios were computed on two time periods: for the first six months after initiation of anticoagulant treatment, and for the following months. This was done to take into account the time-dependent risks of developing an rVTE or a bleeding event, as these events were more likely to occur in the first six months.

**Table 1.** Efficacy and safety inputs of edoxaban vs. warfarin, from the Hokusai-VTE clinical trial.

Parameter	Base case value [DSA range]	
Odds Ratio (OR) of edoxaban vs. warfarin	First six months	Following months
VTE recurrence	0.83 [0.59–1.17]*	0.82 [0.25–2.68]*
CRNMB	0.78 [0.66–0.93]	0.89 [0.59–1.32]*
Non-ICH MB	1.15 [0.75–1.75]*	0.44 [0.13–0.42]
ICH	0.23 [0.07–0.81]	0.39 [0.08–2.02]*
Baseline monthly probabilities (event rate in warfarin arm)	First six months	Following months
VTE recurrence	1.8% [1.4–2.2%]	0.2%[0.0–0.4%]
CRNMB	1.7%[1.4–2.0%]	0.4%[0.2–0.6%]
Non-ICH MB	0.16%[0.07–0.26%]	0.04%[0.00–0.10%]
ICH	0.03%[0.00–0.08%]	0.03%[0.00–0.08%]

*Odds-ratios which were set to 1 in the scenario analysis. DSA: Deterministic sensitivity analysis, OR: Odds-ratio, VTE: Venous Thromboembolism, CRNMB: Clinically relevant non major bleeding, ICH: Intracranial haemorrhage.

As the Hokusai-VTE trial did not show a significant difference in mortality rates following an ICH and non-ICH MB for edoxaban vs. warfarin, the aggregated data, i.e., the mortality rate for OACs combined, was modelled. Whenever the model required data that could not be retrieved from the Hokusai-VTE trial, transition probabilities were derived from publications; these data included the probability of developing CTEPH [10] and PTS [11] after a VTE event, the probability of disability following ICH [12], some disease-specific mortality rates (due to a PE recurrence [13], the probability of dying when disabled from ICH [14], and short-term [15] and long-term mortality [16] from CTEPH), and the probability of developing rVTE while off treatment [17] (Table 2). The general mortality of the cohort depended on the gender distribution and mean age across the time horizon. To account for this gender and age distribution we used UK life tables as source of input in our model [18].

**Table 2.** Inputs for the base case analysis and ranges of values used for the deterministic sensitivity analysis.

Parameter	Base case value [DSA range]
**Clinical parameters**	
Proportion of PE in index events [8]	40.1% [39.1–41.2%]
Proportion of PE with also DVT in index events [8]	44.1% [42.4–45.8%]
Proportion of PE among VTE recurrences [8]	56.5% [50.7–62.4%]
Probability of VTE recurrence while off treatment [10]	0.42% [0.36–0.49%]
Probability of developing CTEPH after a PE [10]	4.8% [2.3–9.6%]
Probability of developing PTS after a DVT [11]	2.7% [2.7–8.1%]
Probability to become disable after ICH event [12]	65% [56–75%]
Death after PE recurrence [13]	6.1% [3.0–30.8%]
Death after non-ICH MB [8]	6.1% [1.4–10.8%]
Death after ICH [8]	26.1% [8.1–44.0%]
Death after pulmonary endarterectomy (CTEPH) [15]	4.4% [2.6–6.2%]
Long-term monthly mortality post-ICH [14]	3.3% [1.4–3.3%]
Long-term monthly mortality post-CTEPH [16]	0.7% [0.6–0.7%]
**Utility parameters**	
Utility PE [20]	0.67 [0.30–0.72]
Utility DVT [20]	0.71 [0.54–0.80]
Utility decrement with warfarin (vs. edoxaban) [21]	1.4% [0.0–1.9%]
Utility decrement due to CRNMB [22]	5% [0–10%]
Utility decrement due to Non-ICH MB [22]	32% [9–48%]
Utility decrement due to ICH [22]	65% [44–85%]
Utility decrement due to disability following ICH [22]	65% [44–85%]
Utility decrement due to CTEPH [23]	30% [26–34%]
Utility decrement due to PTS [22]	14% [0–31%]
**Resource use & economic parameters**	
*Anticoagulant treatment and heparin*	
Warfarin monthly costs (£) (eMIT)	1.22
Edoxaban monthly costs (first/subsequent) (£)	44.5/53.3
Heparin daily cost (incl. administration costs) (£) (BNF)	12.8 [7.9–15.5]
Days of heparin lead-in with warfarin/edoxaban	6.5 [5.0–8.5]/5.0 [5.0–7.5]
*INR Monitoring while on warfarin*	
Cost INR visit First/Subsequent (£) [24]	87 [42–92]/26 [16–37]
INR visits for titration	4.0 [3.0–6.0]
Monthly INR visits (after 1st month)	1.0 [0.8–1.7]
*VTE event costs*	
Costs per PE event (£) [24]	1,647 [1,238–3,668]
Cost per DVT event [24]	551 [654–1,086]
*Costs due to Bleeding Complications (£)*	
Monthly costs for disabled ICH	524 [164–1,053]
Inpatient cost due to ICH [24]	3,012 [1,964–6,493]
Non-ICH MB (inpatient) [24]	2,940 [2,330–5,610]
CRNMB (inpatient + outpatient) [24]	384 [308–461]
*CTEPH & PTS*	
Monthly PTS costs (First/Subsequent) (£) [24]	168 [167–173]/23 [23–24]
Cost of Pulmonary endarterectomy (£) [24]	7,824 [6,540–10,227]
% CTEPH patients undergoing endarterectomy [28]	50% [40–60%]
Monthly drug costs (£) (BNF)	1,348[1,078–1,617]

DSA: Deterministic sensitivity analysis, PE: Pulmonary embolism, DVT: Deep venous thrombosis, VTE: Venous thromboembolism, CTEPH: Chronic thromboembolic pulmonary hypertension, PTS: Severe post-thrombotic syndrome, MB: Major bleeding, CRNMB: Clinically relevant non major bleeding, INR: International normalised ratio, BNF: British National Formulary.

### Health utilities

A literature review was performed to obtain the utility values associated with VTE (both DVT and PE), PTS and CTEPH, treatment related (dis)utility values, and the (dis)utility associated with potential adverse events. The baseline utility levels for the general population were obtained from the landmark national EQ-5D survey reported by Kind et al. [19]. These utility values were used as a basis to estimate the utility values of each health state in the present model.

The baseline utility values for DVT and PE estimated by Cohen et al. [20] were weighted based on the proportion of the cohort experiencing DVT and PE, resulting in a utility value of 0.69 for iVTE, and an estimated decrement of 14% for the first month following an rVTE [20]. A relative utility decrement of 1.37% was associated with warfarin treatment compared to edoxaban treatment, as warfarin treatment is known to lightly impact the quality of life due to the frequent INR monitoring, and the numerous food and drug interactions [21]. These discomforts are generally not experienced by patients treated with edoxaban. Utility decrements applied for CRNMB, non-ICH MB, ICH, and disability post-ICH were 5.00%, 31.58%, 65.26%, and 65.26% respectively. Utility value decrements of 13.68% and 30.00% were applied for every case of PTS [22] and CTEPH [23] respectively, for all subsequent cycles. For the few patients with both PTS and CTEPH, the utility decrements were taken into account multiplicatively.

### Resource use and costs

The model included resource utilisation, drug prices, and the costs of events and health states. All costs were obtained from NHS reference costs 2015/16 [24] (the latest available at the time of the study) unless stated otherwise. The costs of the heparin lead-in, for both edoxaban and warfarin, were solely applied to the first cycle of treatment period following both the index event and recurrence. The drug prices were obtained from the British National Formulary (BNF) and from the NHS Electronic Drug Tariff. Assuming equal use of all heparins, the daily heparin cost was estimated as the average cost of the four different heparin products available in the UK and combined with the weighted average of self-administration costs, and the cost of administration by a professional. The administration costs applied for a limited number of days and were based on the assumption that 87% of patients will self-administer LMWH (i.e., with no administration cost) [24]. The daily cost of warfarin was estimated to be £0.04 and the LMWH/heparin lead-in is 6.5 days for warfarin and 5 days for edoxaban. Subsequently, the total cost related to LMWH/heparin acquisition and administration was £64.10 for the edoxaban arm, and £83.33 for the warfarin arm.

Since warfarin has a narrow therapeutic index, repeated measurements of INR are required during the course of treatment. The cost of treatment in the warfarin arm, was £273.81 for the first cycle, 23.5 days of treatment, (including INR monitoring and 6.5 day concomitant heparin), and £26.73 for the following cycles, 30 days of treatment, (including INR monitoring). Patients on edoxaban received a 5 day heparin lead-in. The daily cost of edoxaban was assumed to be £1.75. Since INR monitoring is not required for NOACs like edoxaban, the treatment costs in the edoxaban arm included the drug acquisition costs for edoxaban and heparin, and was estimated to be £108.62 during the first cycle, 25 days of treatment with edoxaban (including heparin), and £53.27 for the following cycles, 30 days of treatment with edoxaban.

The costs of VTE recurrence included hospitalisation costs [25], costs related to diagnosis and treatment of DVT (£551) and PE (£1,647, resulting in an average cost of £1,236 per VTE recurrence. The cost of a major bleed included the hospital admission costs. Accordingly, the cost considered for ICH was £3,012, and that for non-ICH MB was £2,940 [26]. Costs associated with CRNMB were estimated to be £384 [26].

The PTS related costs included vascular surgery during the first visit, and the costs related to the follow-up visits. The cost for the first month was estimated to be £168, corresponding to a first appointment for vascular surgery, and the average cost for each of the following months was estimated to be £23 per month, assuming 2 follow-up visits per year [27]. The costs related to CTEPH included treatment costs, surgery and drug therapy. Assuming that 50.3% of CTEPH patients required pulmonary endarterectomy [28] with a unit cost of £7,824, and follow-up costs of £1,348 per month, the total average cost for CTEPH treatment was estimated at £5,285 for the first month, and £1,348 for the following months. The long-term impact of ICH resulting in disability was categorised as mild, moderate, and severe for both the care centre costs and home care costs. On average, the monthly costs for a disabled patient with ICH were estimated to be £524 [12,29]. It was assumed that there were no costs associated with death and the off treatment health state.

### Base case, sensitivity analyses, scenario analyses and subgroup analyses

The base case analysis evaluated the costs and health outcomes of edoxaban vs. warfarin for adults with an index DVT and/or an index PE (index DVT ± PE) over a lifetime horizon, assuming an initial treatment duration of 6 months.

Sensitivity analyses were performed to evaluate the impact of assumptions used in the model, and to assess the variability surrounding model inputs. To identify key drivers of the model, a univariate deterministic sensitivity analysis was performed on all model parameters associated with uncertainty. In this analysis, one parameter at a time was varied using lower and upper bounds (Table 1). Those results are shown as a tornado diagram. A multivariate probabilistic sensitivity analysis was also performed to estimate the effect of overall uncertainty in the evaluation through repeated sampling of parameter values set to follow appropriate statistical distributions. Two thousand simulations were generated and the model was run for each set of parameters, providing an estimate of the variability of results. The results were presented by an incremental cost-effectiveness plane and cost-effectiveness acceptability curves (CEACs).The following scenario analyses were also investigated: reduced time horizon to 1 year and 5 years and non-significant odds ratios set to 1.

Finally, the cost-effectiveness of edoxaban vs. warfarin was analysed for four population subgroups depending on the nature of the iVTE: patients with index DVT but no index PE (Index DVT only), patients with index PE with or without DVT (Index PE (± DVT)), patients with index PE but no index DVT (Index PE only), and patients with both index DVT and index PE (Index PE + DVT).

## Results

### Base case

The results of the base case analysis are summarised in Table 3. The cumulative incidences of rVTE, PTS, and CTEPH were similar in both edoxaban and warfarin arms. Yet, the cumulative incidence of adverse events (CRNMB, non-ICH MB and ICH MB) was lower in the edoxaban arm. As a consequence of fewer MBs in the edoxaban arm, mortality was also lower. These results translated into an improvement of health outcomes, with 0.029 incremental life years and 0.033 incremental QALYs gained, compared to warfarin. Treatment-related costs for people treated with edoxaban were higher while costs related to adverse events were lower due to fewer bleeds experienced by patients treated with edoxaban. The cumulative benefits related to edoxaban vs. warfarin (0.033 QALY) were associated with lower total costs for edoxaban (-£55 versus warfarin) resulting in edoxaban being dominant when compared to warfarin. Assuming a threshold of £20,000 per QALY, the corresponding NMB was £717 (Confidence interval: [£398; £960]).

**Table 3.** Model results for base-case and subgroups analysis.

	1. Base case analysis, VTE population		2. Subgroup analyses							
			(a) Index DVT only		(b) Index PE (± DVT)		(c) Index PE – DVT		(d) Index PE + DVT	
	Edoxaban	Warfarin	Edoxaban	Warfarin	Edoxaban	Warfarin	Edoxaban	Warfarin	Edoxaban	Warfarin
**Clinical outcomes (cumulative incidence over the time horizon)**										
VTE recurrence	105.53%	105.56%	124.42%	124.20%	83.77%	84.04%	86.15%	86.42%	78.54%	78.84%
CRNMB	13.85%	15.53%	13.51%	15.38%	14.63%	16.02%	16.41%	18.38%	11.41%	11.50%
Non-ICH MB	4.74%	4.62%	2.09%	2.15%	3.88%	3.50%	5.29%	4.62%	1.12%	1.32%
ICH MB	1.83%	2.09%	0.45%	0.74%	3.84%	4.07%	2.94%	3.25%	5.55%	5.66%
PTS	4.07%	4.07%	4.09%	4.08%	2.22%	2.22%	0.55%	0.55%	4.84%	4.85%
CTEPH	4.70%	4.70%	3.45%	3.45%	7.54%	7.55%	7.93%	7.94%	7.06%	7.06%
Specific Death	4.17%	4.29%	2.38%	2.51%	7.40%	7.51%	7.26%	7.40%	7.81%	7.87%
**Health outcomes**										
Life years	16.523	16.494	16.678	16.645	16.169	16.143	16.141	16.109	16.186	16.170
**QALYs**	1.846	1.842	0.546	0.538	3.299	3.321	3.238	3.259	3.387	3.414
On treatment	10.703	10.677	12.347	12.322	8.696	8.642	8.732	8.674	8.623	8.571
Off treatment	0.040	0.040	0.046	0.046	0.034	0.034	0.034	0.034	0.032	0.032
Recurrent VTE	0.008	0.004	0.006	0.005	0.013	0.000	0.014	0.003	0.011	−0.006
Adverse Events	−0.101	−0.099	−0.082	−0.081	−0.119	−0.117	−0.102	−0.101	−0.148	−0.146
Complications (PTS & CTEPH)	1.846	1.842	0.546	0.538	3.299	3.321	3.238	3.259	3.387	3.414
Total QALYs	12.497	12.464	12.862	12.831	11.923	11.880	11.917	11.870	11.905	11.865
**Costs**										
Treatment costs (excluding INR monitoring)	£392	£90	£392	£90	£392	£90	£391	£90	£394	£90
INR monitoring	£0	£323	£0	£323	£0	£323	£0	£323	£0	£324
Recurrent VTE (acute & treatment)	£2,045	£2,049	£1,252	£1,250	£3,063	£3,091	£3,101	£3,128	£3,016	£3,048
CRNMB	£71	£77	£39	£46	£131	£137	£123	£131	£147	£149
Non ICH MB	£92	£89	£51	£53	£82	£70	£109	£89	£27	£33
ICH (acute and long term costs)	£98	£124	£33	£62	£197	£220	£150	£180	£286	£295
Complications (PTS & CTEPH)	£4,439	£4,439	£2,431	£2,427	£7,985	£7,994	£8,168	£8,177	£7,785	£7,797
Total costs	£7,136	£7,191	£4,198	£4,252	£11,850	£11,925	£12,042	£12,118	£11,656	£11,736
**Cost-effectiveness analysis**										
Incremental QALYs	0.033		0.031		0.043		0.046		0.046	
Incremental costs	-£55		-£54		-£74		-£76		-£81	
Cost per QALY gained (ICER)	Dominant		Dominant		Dominant		Dominant		Dominant	
Net Monetary Benefit	£717		£680		£939		£1,005		£880	

PE: Pulmonary embolism, DVT: Deep venous thrombosis, VTE: Venous thromboembolism, CTEPH: Chronic thromboembolic pulmonary hypertension, PTS: Severe post-thrombotic syndrome, MB: Major bleeding, CRNMB: Clinically relevant non major bleeding, INR: International normalised ratio, ICER: Incremental cost-effectiveness ratio.

### Sensitivity analyses

#### Deterministic sensitivity analysis

**Figure 2.**
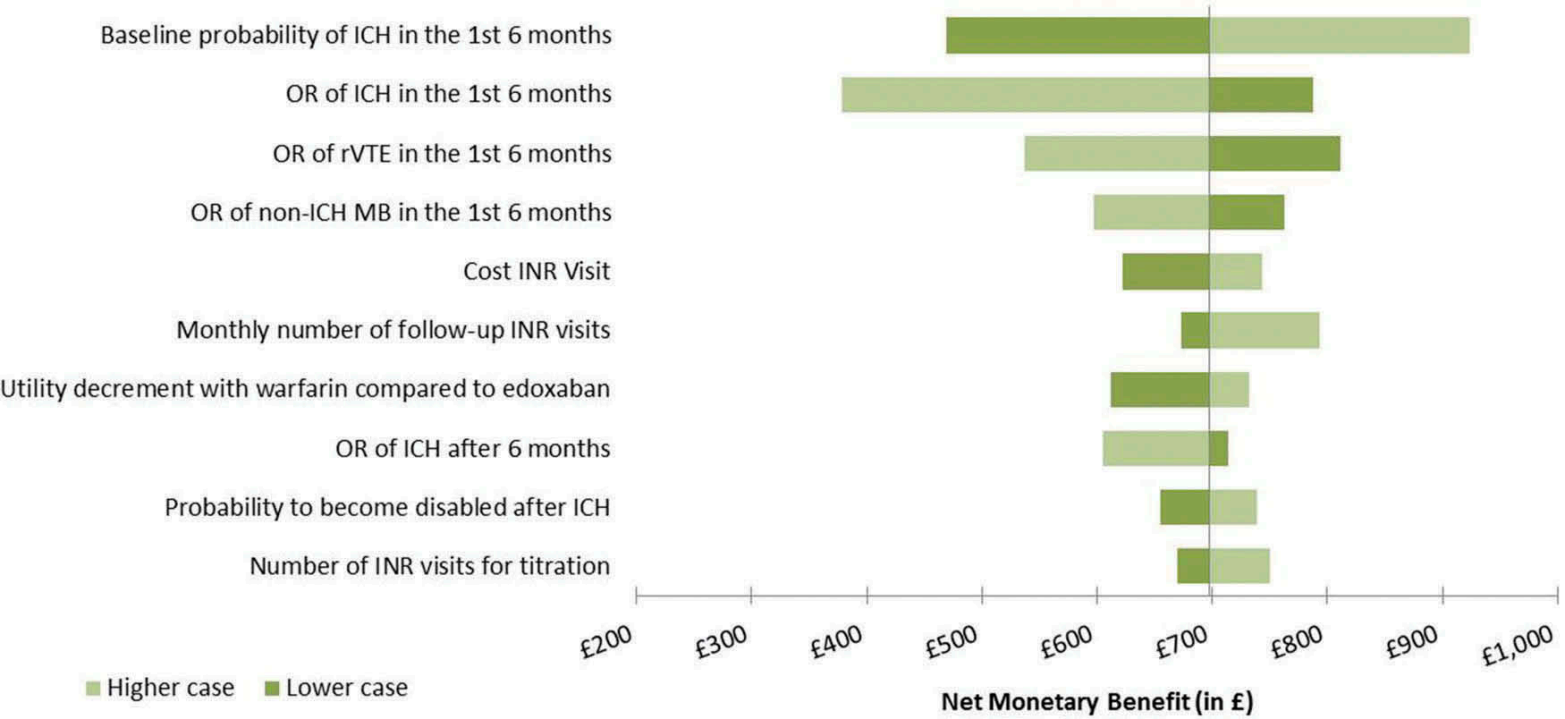
Deterministic sensitivity analysis of the cost effectiveness of edoxaban vs. warfarin (the 10 most impactful parameters are shown).

Deterministic sensitivity analyses were conducted on the NMB, and the 10 main drivers of the analyses are illustrated in the tornado diagram (Figure 2). The main driver was the probability of occurrence of ICH with a NMB ranging from £487 to £947. The other important drivers of the analysis were the probability of occurrence of rVTE and non-ICH MB between edoxaban and warfarin, and inputs related to the INR monitoring in the warfarin arm (number and cost of the INR visits). When the assumed disutility of 1.4% associated with the use of warfarin to reflect the added burden of monitoring visits on the patient’s quality of life was removed, the NMB dropped to £694, against £717 in the base case. In all cases explored in the deterministic sensitivity analyses, edoxaban remained cost effective compared to warfarin when considering a £20,000 willingness-to-pay per QALY.

#### Probabilistic sensitivity analysis

**Figure 3.**
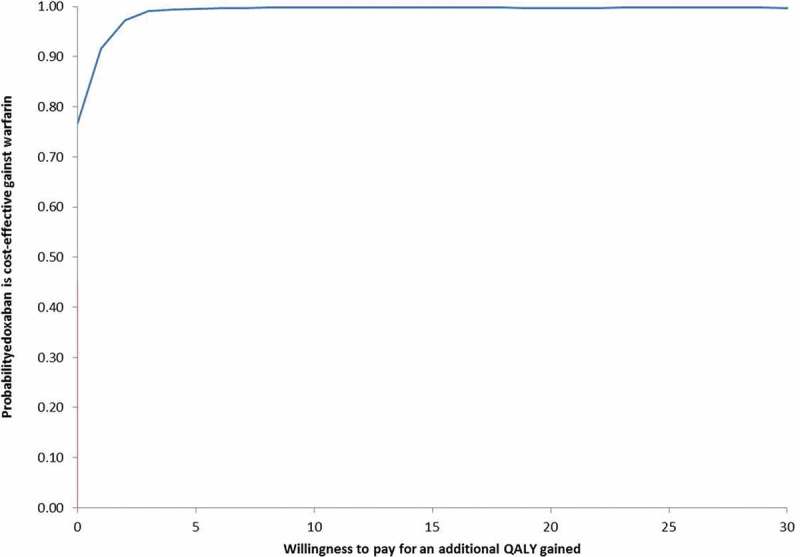
Incremental cost-effectiveness plane (a) and cost-effectiveness acceptability curve (b) of edoxaban vs. warfarin (n = 2,000 simulations).

Over the 2,000 simulations of the probabilistic sensitivity analysis, edoxaban was dominant (less costs and more QALYs) in 88.6% of cases (Figure 3(a)). Moreover, when considering a £20,000 willingness-to-pay per QALY, edoxaban was cost-effective vs. warfarin in 99.5% of cases (Figure 3(b)).

**Figure:**
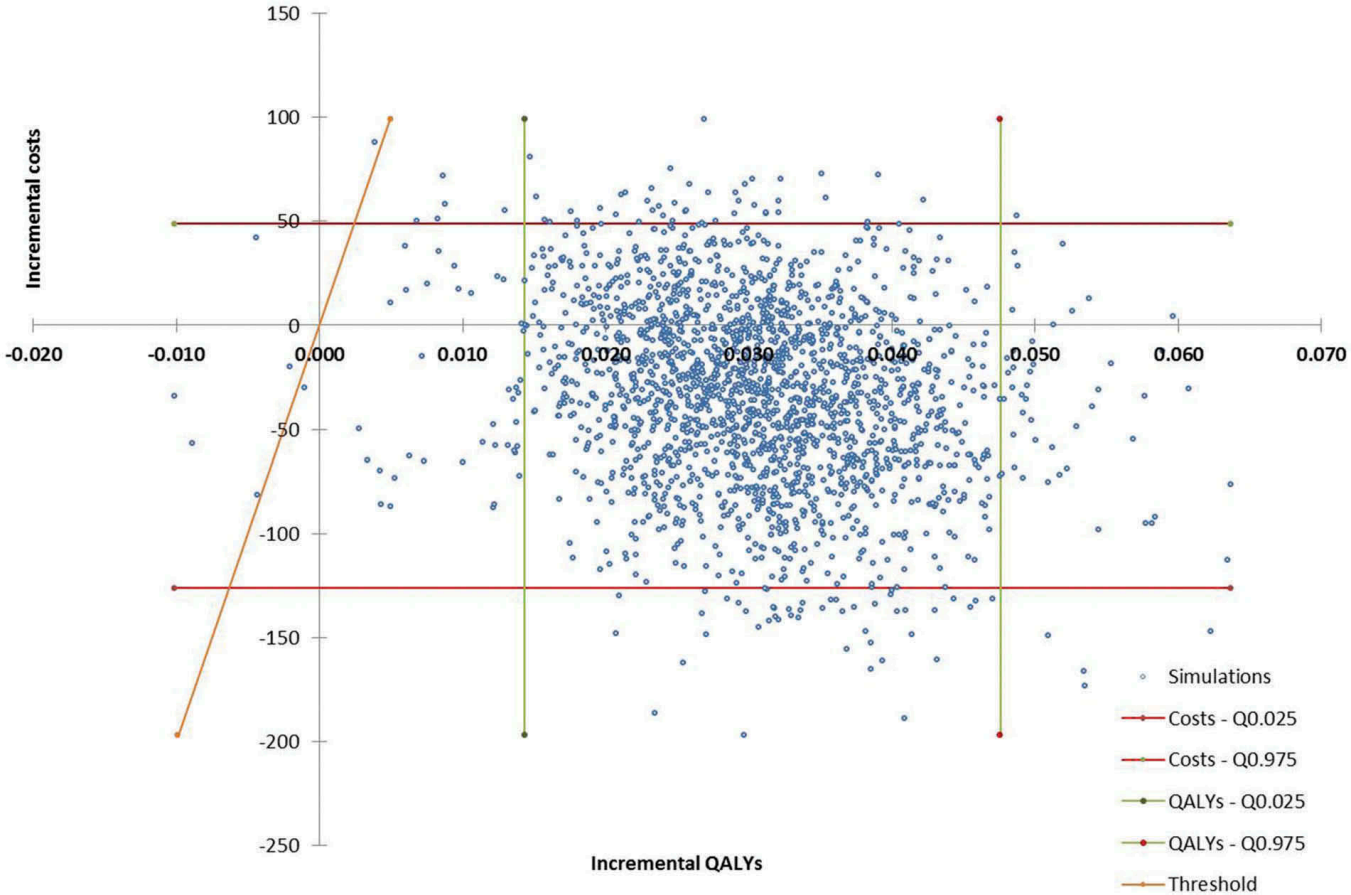

### Scenario analyses

When the non-significant odds ratio was set to 1, edoxaban remained dominant compared to warfarin, but with a smaller difference: £41 of savings and 0.03 additional QALYs with edoxaban compared to warfarin. With a reduced time horizon, edoxaban was still found to be dominant, with £42 and 0.007 additional QALYs and £56 and 0.012 additional QALYs with a 1-year and 5-year time horizon respectively.

### Subgroup analyses

The results of the subgroup analyses (Table 3) were similar to the results obtained in the base case analysis. In the four subgroups, the incidence of rVTE was similar in both edoxaban and warfarin arms. Edoxaban was also associated with fewer adverse events, except for non-ICH MBs in the PE without DVT subgroup where there were fewer events in the warfarin arm (3.93% vs. 3.42%) although these rates were associated with high uncertainty (OR [95%CI]: 1.74 [0.82–3.70]) due to a small number of events observed in each arm of the Hokusai-VTE trial. The costs saved with edoxaban were higher in the three subgroups including patients with index PE than in the base case (-£74 in the PE with or without DVT subgroup, -£76 in the subgroup with index PE without DVT and -£81 for patients with both index PE and index DVT) due to lower cost differences attributed to adverse events, while incremental QALYs were above 0.04. Results were slightly less in favour of edoxaban in the index DVT only subgroup with more VTE recurrences in the edoxaban arm than in the warfarin arm, compensated by fewer bleeds. The incremental QALYs and costs were 0.031 and -£54 in the latter subgroup.

## Discussion

The current analyses evaluated the cost-effectiveness of edoxaban in comparison to warfarin for the treatment of VTE for the UK setting from an NHS perspective. Edoxaban was consistently associated with greater QALYs and lower costs than warfarin and is therefore dominant when compared to warfarin, although the cost difference was not substantial. Subgroup analyses confirm the results to be consistent with the general VTE population with edoxaban being dominant (lower costs and higher QALYs gained) for all the subgroup analyses. Sensitivity analyses revealed that the findings of the base case analysis were robust despite variations in the inputs. The main driver of the model is the probability of an ICH occurrence and the incidence of ICH is likely to be higher in patients not included in clinical trials. The scenario analyses with non-significant OR set to 1 confirmed the robustness of the base case results as well as the scenarios with a shorter time horizon.

Another cost-effectiveness study of edoxaban for the treatment of VTE in adults in comparison to warfarin was performed in the USA. The model was developed using patient-level data from the Hokusai-VTE trial, with clinical events costs from a real-world database. Edoxaban was found to be a cost-effective alternative to warfarin in VTE patients in the USA with an ICER of $22,057 per QALY [30]. The high ICER in the US study compared to our study partly resided in a greater difference in the daily drug costs between edoxaban and warfarin ($9.24 vs. $0.36 in the US, against £1.75 vs. £0.04 in the UK).

Other NOACs have been approved in the UK for the treatment and secondary prevention of venous thromboembolism, namely apixaban, dabigatran and rivaroxaban. We did not include these molecules in the analysis for two main reasons. First, the standard of care in the UK remains treatment with a VKA such as warfarin, thus it is the principal comparator for edoxaban. Second, evidence regarding potential differences in efficacy and safety between the NOACs is scarce, as there is still no direct comparison available. Several indirect treatment comparisons have been performed [31–33], indicating similar results overall. Apixaban was found to have a comparative advantage over the other NOACs in terms of bleed occurrences, although there has been high uncertainty around these results, the clinical trials of the NOACs being substantially different in their designs. However, as the other NOACs are likely to become the standard of care, further investigations comparing all the NOACs together should be performed in the future.

The current model has a comprehensive structure which includes eleven different health states, encompassing all the relevant (adverse) events and complications that a patient with VTE treated with oral anticoagulants might encounter. The life-time horizon assures the inclusion of both the short term as well as the long term consequences of VTE, such as PTS and CTEPH. Our model is similar to previously validated, published models in this therapeutic area [34], while allowing greater flexibility in terms of allowing for various treatment durations, and analysis of different subgroups. The model takes into account the current recommendations by treating VTE recurrence longer than the first event (6 months for first event in the base case, from 6 months to long term treatment after a VTE recurrence depending on the type of recurrence). The model accounts for time-varying event rates (first 6 months of treatment vs. following months). Therefore, the model allows for a more accurate prediction of cumulative events as the (adverse) event rates and complications related to VTE are more likely to occur during the first six months. Finally, the model relies on robust clinical data mainly stemming from a clinical trial designed to reflect clinical practice.

However, the model has also several limitations. Indeed, the VTE patient population is heterogeneous, resulting in a broad spectrum of treatment strategies for the related subgroups. To keep the model simple and transparent, a number of assumptions were made. First, all patients were assumed to be treated for the same initial treatment period (irrespective of the VTE type; DVT or PE). The model did not distinguish between provoked and unprovoked VTE as the HOKUSAI study was able to enrol both types of patients. Patients with provoked VTE are treated for a shorter period of time since they have a lower risk of VTE recurrence.

Second, all patients experiencing a major bleed were assumed to discontinue anticoagulation therapy until the potential for next VTE recurrence appears. Stopping anticoagulant treatment is in line with clinical practice: the priority being to reverse bleeding, so continued anticoagulation would be inappropriate. We accept that some patients would recommence anticoagulation after a major bleed, particularly in the first 2 months following the diagnosis of VTE. These patients would be at higher risk of a bleeding recurrence. However, it is not clear what would happen after a VTE recurrence following a previous major bleed. This remains a relatively rare situation.

Third, DVT and PE were modelled within the same health state. Although some other models considered separate states for PE and DVT, this approach seemed appropriate due to the lack of detailed data regarding the long-term sequence of recurrences for PE and DVT. The model takes the proportion of DVT and PE occurrences into account in the weighted average of related costs and probabilities of the disease related complications, PTS and CTEPH.

Finally, there were uncertainties regarding some inputs used in the model. For instance, the true costs of INR monitoring are currently highly debated in the literature [35]. Conservative estimates were taken into account in the base case, while uncertainty around these costs was considered in the sensitivity analyses. The sensitivity analyses showed that variations regarding these parameters were not likely to impact the conclusion of this analysis.

For future investigation it will be of huge interest to perform a cost-effectiveness analysis including all the NOACs and using real-world data instead of clinical trial data to feed the model.

## Conclusion

Based on data from the Hokusai-VTE trial and relevant publications used to populate the model, this analysis suggests that edoxaban represents a valuable alternative compared to warfarin for the treatment of patients with VTE in the UK.

## Notes

EC and AJ are employees of Creativ-Ceutical and received funding from Daiichi Sankyo Europe GmbH for this study. PG is an employee of Daiichi Sankyo Europe GmbH. PV, BVH and AC received consulting fees from Daiichi Sankyo Europe GmbH, Bayer, Jannsen, Boehringer Ingelheim, Pfizer, Portola and Bristol-Myers Squibb.
